# What Is the Most Bothersome Lower Urinary Tract Symptom? Individual- and Population-level Perspectives for Both Men and Women

**DOI:** 10.1016/j.eururo.2014.01.019

**Published:** 2014-06

**Authors:** Arnav Agarwal, Leyla N. Eryuzlu, Rufus Cartwright, Kristian Thorlund, Teuvo L.J. Tammela, Gordon H. Guyatt, Anssi Auvinen, Kari A.O. Tikkinen

**Affiliations:** a Department of Clinical Epidemiology and Biostatistics, McMaster University, Hamilton, ON, Canada; b Faculty of Health Sciences, McMaster University, Hamilton, Ontario, Canada; c Department of Epidemiology and Biostatistics, Imperial College London, London, UK; d Department of Urogynaecology, St. Mary's Hospital, London, UK; e Stanford Prevention Research Center, Stanford University, Stanford, CA, USA; f Department of Urology and School of Medicine, Tampere University Hospital and University of Tampere, Tampere, Finland; g Department of Medicine, McMaster University, Hamilton, Ontario, Canada; h School of Public Health, University of Tampere, Tampere, Finland; i Department of Urology, Helsinki University Central Hospital and University of Helsinki, Helsinki, Finland

**Keywords:** Age factors, Bothersomeness, Definition, Lower urinary tract symptoms, Overactive bladder, Prevalence, Sex, Urinary incontinence, Urination disorders, Voiding dysfunction

## Abstract

**Background:**

No study has compared the bothersomeness of all lower urinary tract symptoms (LUTS) using a population-based sample of adults. Despite this lack of evidence, investigators have often cited their LUTS of interest as the “most bothersome” or “one of the most bothersome.”

**Objective:**

To compare the population- and individual-level burden of LUTS in men and women.

**Design, setting, and participants:**

In this population-based cross-sectional study, questionnaires were mailed to 6000 individuals (18–79 yr of age) randomly identified from the Finnish Population Register.

**Outcome measurements and statistical analysis:**

The validated Danish Prostatic Symptom Score questionnaire was used for assessment of bother of 12 different LUTS. The age-standardized prevalence of at least moderate bother was calculated for each symptom (population-level burden). Among symptomatic individuals, the proportion of affected individuals with at least moderate bother was calculated for each symptom (individual-level bother).

**Results and limitations:**

A total of 3727 individuals (62.4%) participated (53.7% female). The LUTS with the greatest population-level burden were urgency (7.9% with at least moderate bother), stress urinary incontinence (SUI) (6.5%), nocturia (6.0%), postmicturition dribble (5.8%), and urgency urinary incontinence (UUI) (5.0%). Burden from incontinence symptoms was higher in women than men, and the opposite was true for voiding and postmicturition symptoms. At the individual level, UUI was the most bothersome for both genders. Although the response proportion was high, approximately a third did not participate.

**Conclusions:**

Both men and women with UUI report moderate or major bother more frequently than individuals with other LUTS. At the population level, the most prevalent bothersome symptoms are urgency, SUI, and nocturia.

**Patient summary:**

Urinary urgency was the most common troubling symptom in a large population-based study; however, for individuals, urgency incontinence was the most likely to be rated as bothersome.

## Take Home Message

Population-based study assessing bother of individual lower urinary tract symptoms (LUTS) found the highest age-standardized prevalence for bothersome urgency. However, among individuals affected by LUTS, urgency incontinence was the most likely to cause significant bother. LUTS were generally well tolerated across ages and genders.

## 1 Introduction

Lower urinary tract symptoms (LUTS) are divided into three groups: storage symptoms (daytime urinary frequency, nocturia, urinary urgency, and urinary incontinence), voiding symptoms (slow stream, splitting or spraying, intermittent stream, hesitancy, straining, and terminal dribble), and postmicturition symptoms (feeling of incomplete emptying and postmicturition dribble) [1]. These symptoms have a major impact on health-related quality of life [2] and are associated with substantial personal and societal expenditures [3]. Because LUTS are associated both with age and obesity [4], [5], the population burden of these conditions is likely to increase with future demographic shifts.

Symptom bother increases as the number of symptoms increases and as symptoms become more severe [6]. However, the symptom impact is not well defined by their frequency alone [3]. Assessment of the bother of symptoms is of key clinical relevance because it relates to both quality-of-life impairment and treatment seeking [7], [8]. Consequently, clinical practice guidelines suggest that only those reporting bothersome symptoms should be targeted for intervention, and watchful waiting is appropriate for those with minimal symptoms [3], [9].

Investigators have often described their LUTS of interest as the “most bothersome” or “one of the most bothersome” [10], [11], [12], [13]. Despite these claims, very few studies have evaluated the relative degree of bother across all LUTS, and none have attempted to establish the most bothersome LUTS in a population-based study of both genders and all adult ages [14], [15], [16], [17]. From the population perspective, the symptom with the greatest burden is the most prevalent bothersome symptom. From the individual perspective, the symptom with the greatest burden is simply the symptom most likely to be rated as bothersome among the affected. Thus the purpose of this analysis is to compare the degree of bother of major LUTS in both men and women 18–79 yr of age from both the population and individual perspectives.

## 2 Methods

### 2.1 The Finnish National Nocturia and Overactive Bladder Study

The Finnish National Nocturia and Overactive Bladder Study cohort comprises a population-based sample of men and women 18–79 yr of age in Finland. Its aim is to assess the prevalence, natural history, impact, and risk factors of urinary symptoms at the population level. Detailed study procedures have been published [18], [19], [20]. Briefly, in 2003–2004, questionnaires were mailed to individuals randomly identified from the Population Register Centre of Finland. Stratification by age was used in individual selection with oversampling of younger age groups to ensure an adequate number of individuals with urinary storage symptoms in each age group [18]. The questionnaire gathered information on frequency and bother of LUTS, quality of life, numerous conditions and medications, as well as information on sociodemographic, anthropometric, and reproductive factors. Exemption from ethical review was granted by the ethics committee of the Pirkanmaa Hospital District (Tampere, Finland), as permitted by the Finnish regulations on surveys. The reporting of this analysis conforms to the Strengthening the Reporting of Observational Studies in Epidemiology statement (www.strobe-statement.org).

### 2.2 Measures

The frequency and bother of LUTS were assessed using the questions from the Danish Prostatic Symptom Score (DAN-PSS; Supplemental Table 1) [21], which are consistent with the International Continence Society definitions [1]. The DAN-PSS includes assessment of 12 LUTS: hesitancy, weak stream, incomplete emptying, straining, daytime frequency, nocturia, urgency, urgency urinary incontinence (UUI), pain/burning, postmicturition dribble, stress urinary incontinence (SUI), and other incontinence. Occurrence of most LUTS was measured on a 4-point scale consisting of *never, rarely, often*, and *always* (Supplemental Table 1). Bother of all LUTS was reported on a 4-point scale: *none, small, moderate*, or *major* (Supplemental Table 1). Among symptomatic individuals (those who reported a symptom occurring at least rarely or equivalent were considered symptomatic; see response options in Supplemental Table 1), the age-standardized proportion of individuals with moderate or major bother for each LUTS was calculated to determine the individual level of bother of each symptom. To determine the most prevalent bother at the population level, the age-standardized prevalence of respondents with moderate or major bother was calculated for each symptom.

### 2.3 Statistical analysis

Subjects were stratified into age groups: 18–29, 30–39, 40–49, 50–59, 60–69, and 70–79 yr. Confidence Interval Analysis v.2.1.2 software (Trevor Bryant, University of Southampton, UK) was used for calculating age-standardized prevalence and confidence intervals (CIs). Other analyses were performed with SPSS v.16.0.0 (IBM Corp., Armonk, NY, USA). Age-standardized prevalence was based on the population structure of Finland in early 2004. Among symptomatic individuals and separately for both genders, the effect of age (classified as 18–39, 40–59, 60–79 yr) on bother (moderate or major vs no or small bother) was assessed using the chi-square test for data stratified by symptom occurrence (rarely, often, always).

## 3 Results

**Fig. 1:**
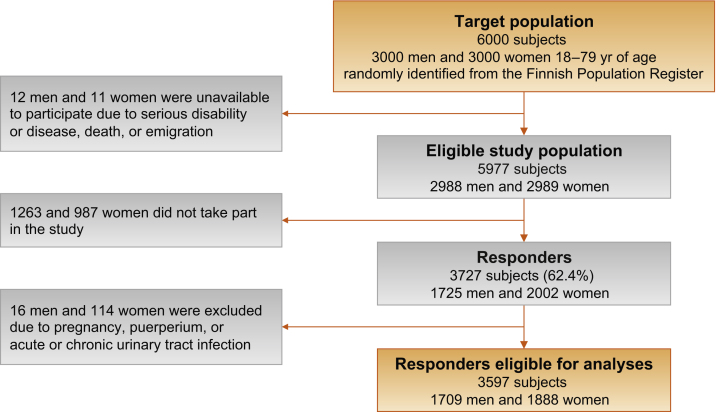
Study flowchart.

Of the 6000 men and women approached, 3727 (62.4%) participated. Of them, 130 were excluded, resulting in 3597 respondents included in the analyses (1709 men and 1888 women) (Fig. 1). The median percentage of participants who provided information on both occurrence and bother for each of the 12 symptoms was 95.5% (range: 91.1–97.7%) for men and 94.3% (range: 87.9–97.5%) for women. For more characteristics of the respondents, see Table 1.

**Table 1:** Age distribution and demographic characteristics among the 1709 men and 1888 women included

Characteristics	Crude *n* (%)	
	Men	Women
Age groups, yr		
18–39	814 (47.6)	919 (48.7)
40–59	525 (30.7)	612 (32.4)
60–79	370 (21.7)	357 (18.9)
Marital status*		
Single	382 (22.4)	368 (19.5)
Married/living together	1214 (71.0)	1256 (66.5)
Divorced/separated	79 (4.6)	145 (7.7)
Widowed	23 (1.3)	106 (5.6)
Education*		
Basic level	473 (27.7)	461 (24.4)
Vocational school	577 (33.8)	574 (30.4)
College	324 (19.0)	453 (24.0)
University	327 (19.1)	376 (19.9)
Employment*		
Student	172 (10.1)	255 (13.5)
Employed	1013 (59.3)	1102 (58.4)
Unemployed	110 (6.4)	131 (6.9)
Retired	389 (22.8)	375 (19.9)
Urbanity*		
Nonurban	1050 (61.4)	1114 (59.0)
Urban	648 (37.9)	764 (40.5)
Body mass index, kg/m^2^*		
<25	697 (40.8)	1061 (56.2)
25–30	759 (44.4)	537 (28.4)
30–40	207 (12.1)	199 (10.5)
≥40	14 (0.8)	25 (1.3)

* Information on marital status, education, employment, urbanity, and body mass index was available for these percentages of men (women), respectively: 99.4% (99.3%), 99.5% (98.7%), 98.5% (98.7%), 99.4% (99.5%), and 98.1% (96.5%).

**Fig. 2:**
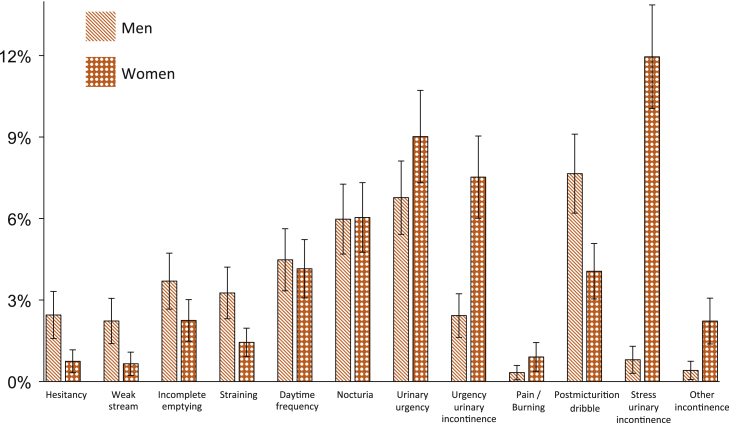
Population perspective: age-standardized prevalence (percentage) of at least moderate bother from lower urinary tract symptoms among men and women. Error bars represent 95% confidence intervals.

From the population perspective, after age standardization, the most frequent at least moderately bothersome LUTS was urgency (7.9%), followed by SUI (6.5%), nocturia (6.0%), postmicturition dribble (5.8%), and UUI (5.0%) (Table 2). The population prevalence of bothersome SUI was higher in women (12.0%; 95% CI, 10.0–13.9) than in men (0.8%; 95% CI, 0.3–1.3) (Fig. 2; Supplemental Table 2). Furthermore, the population burden of both UUI and other incontinence was greater in women than in men. There was, however, a higher population-level prevalence of bother in men than women from voiding symptoms (hesitancy, weak stream, and straining) and postmicturition dribble (Fig. 2; Supplemental Table 2).

**Table 2:** Gender-combined and age-standardized (1) prevalence (%) of individuals reporting moderate or major bother from different lower urinary tract symptoms (population perspective) and (2) proportion (%) of individuals reporting moderate or major bother among those affected with the symptom (individual perspective)

Symptom	Age-standardized prevalence/proportion	
	Population perspective, %*	Individual perspective,%†
Hesitancy	1.6	4.6
Weak stream	1.4	16.0
Incomplete emptying	3.0	9.1
Straining	2.3	5.3
Daytime frequency	4.3	16.5
Nocturia	6.0	14.9
Urinary urgency	7.9	13.5
Urgency urinary incontinence	5.0	30.7
Pain/Burning	0.6	2.8
Postmicturition dribble	5.8	13.8
Stress urinary incontinence	6.5	19.8
Other incontinence	1.3	11.9

* The age-standardized prevalence of respondents with moderate or major bother was calculated for each symptom.

† Among symptomatic individuals (those who reported a symptom occurring at least rarely), the age-standardized proportion of individuals with moderate or major bother for each lower urinary tract symptom was calculated.

**Fig. 3:**
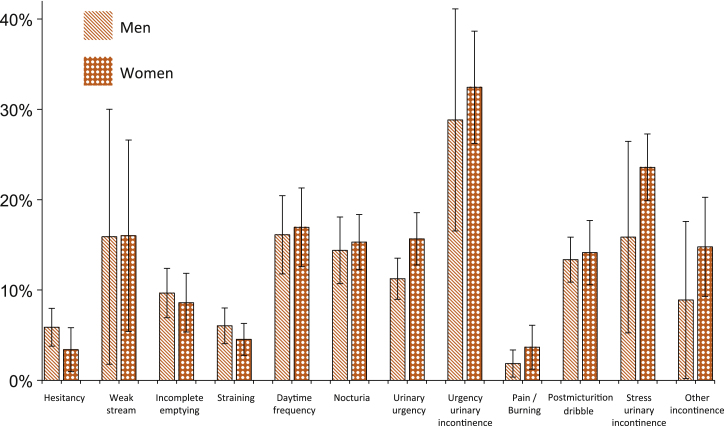
Individual perspective: age-standardized proportion (percentage) of subjects reporting at least moderate bother among symptomatic men and women. Error bars represent 95% confidence intervals.

From an individual perspective, UUI was the symptom most likely to be rated as bothersome, with 30.7% of symptomatic individuals reporting at least moderate bother (Table 2). The degree of bother from all other symptoms was either none or small for at least 80% of symptomatic individuals (Fig. 3; Supplemental Table 3). The CIs of the estimates overlapped between sexes, indicating no major differences in the perceived bother at the individual level between sexes (Fig. 3). In general, no major effect of age on perceived bother was found (Supplemental Table 4). However, *rare* SUI, *rare* UUI, and milder forms of nocturia (one to two times per night) were perceived as less bothersome by older than younger women, and *rare* urgency and milder forms of daytime frequency were perceived as more bothersome for older than younger men (*p* < 0.05 for all; Supplemental Table 5).

## 4 Discussion

We compared the extent to which men and women 18–79 yr of age experienced bothersome LUTS taking into account both population and individual perspectives. Among the 3597 participants, we observed large differences between individual-level and population-level perspectives on symptom bother. “No bother” or “minor bother” were the most common responses reported for every symptom assessed. Urgency was identified as the symptom with the highest prevalence of at least moderate bother at the population level. Among those who experienced a particular symptom, however, UUI was the symptom most likely to be reported as bothersome. Because of its low prevalence, UUI was only the fourth most bothersome LUTS considering the population as a whole. Differences between genders were only observed at a population level. Women more frequently reported bothersome SUI, whereas men more frequently reported voiding and postmicturition symptoms. However, among symptomatic individuals, the distribution of bother was similar in both genders. These findings suggest strongly that although the etiology of LUTS may be different between genders, the bother impact on individuals is similar.

### 4.1 Strengths and limitations

Our data provide a unique comparison of LUTS bother in a population-based study of both genders and wide range of adult ages. Further strengths lie both in the high response and excellent completion rates as well as in the use of age standardization that improves comparability with other studies and generalizability to other populations. The results would have been further bolstered by information about care seeking, but current evidence suggests that only a small proportion of women and men visit their primary care physician with these symptoms [22], [23], [24]. Those individuals with mild symptoms or symptoms with no or small bother are less likely to seek care, highlighting the importance of bother assessment. The Finnish population has low ethnic diversity, but results are likely generalizable to other populations of European descent with a similar socioeconomic structure.

### 4.2 Comparison with other studies

We conducted a PubMed search for English-language articles (published as of December 18, 2013) focused on bothersomeness of LUTS (for further details, see Table 3). Although numerous articles have been published on LUTS [2], [6], [14], [15], [16], [17], [22], [25], [26], [27], much fewer have also directly measured the bother from individual urinary symptoms [14], [15], [16], [17], [25], [26], [27], and only a few of these studies were population based [14], [15], [16], [17]. In contrast to the present study, these earlier population-based studies (Table 3) that assessed bother from individual urinary symptoms have typically only reported on a limited number of LUTS [16] or included a limited age range [14], [16], [17]. Most importantly, none of the earlier studies included both genders [14], [15], [16], [17]. Finally, most of the studies [15], [16], [17] did not aim to establish the most bothersome symptom at either the population or individual level but provided data enabling us to calculate the estimates reported in Table 3. However, the heterogeneity in sampling methods and outcome assessments from previous population-based studies likely explain the variability in results. Different symptoms have been identified as most bothersome including urgency, nocturia, and terminal dribbling (Table 3). Among female sufferers, storage symptoms (including incontinence) were most likely to cause problems, whereas among male sufferers, both storage and postmicturition symptoms were most likely to cause problems in the international Epidemiology of LUTS (EpiLUTS) study that evaluated prevalence and bother of numerous LUTS [26], [28]. As many as 88 150 individuals were contacted mainly from consumer panels (self-selected volunteers and cannot be regarded as population based), and a final sample of 30 000 was evaluated. The comparison of the relative adverse impact (individual perspective) of LUTS was similar between (EpiLUTS) and our study. Overall, in our study, urgency was the symptom most frequently found bothersome at the population level. Of those who were symptomatic, individuals reporting UUI most frequently found the symptom at least moderately bothersome.

**Table 3:** Overview of published English-language population-based studies assessing degree of bother from individual lower urinary tract symptoms*

	Leicestershire (United Kingdom) [14]	Bristol (United Kingdom) [15]	Central Sydney (Australia) [16]	Copenhagen and Storstrøms (Denmark) [17]	Finland (present study)
Data collection method	Interviewer-administered questionnaire	Mailed questionnaire	Telephone interview	Mailed questionnaire	Mailed questionnaire
Sample source	General practice patient list	General practice patient list	Telephone registry	Population registry	Population registry
Respondents	423	2075	340	2860	3727
Response proportion, %	65.2	79.8	65.5	71.7	62.4
Sample age, yr	40–70+	19–97	40–80	40, 45, 50, 55, 60	18–79
Questionnaire used	Maine prostatectomy instrument; ICS-BPH (developmental version)	BFLUTS	Modified IPSS	Modified BFLUTS	DAN-PSS
Total number of urinary symptoms with bother reported	18	14	6	12	12
Number of urinary symptoms per category (voiding/storage/postmicturition/other)	6/8/2/2	4/7/1/2	2/3/1/0	2/8/2/0	3/6/2/1
Symptom with greatest bother among the affected, individual level, W	NA	Nocturia^a^	NA	Continuous incontinence^b^	Urgency urinary incontinence^c^
Symptom with most prevalent bother, population level, W	NA	Stress urinary incontinence^b^	NA	Stress urinary incontinence^b^	Stress urinary incontinence^c^
Symptom with greatest bother among the affected, individual level, M	Daytime frequency	NA	Hesitancy^b^	NA	Urgency urinary incontinence^c^
Symptom with most prevalent bother, population level, M	Terminal dribble	NA	Postmicturition dribble^b^, ^d^	NA	Postmicturition dribble^c^

BFLUTS = Bristol Female Lower Urinary Tract Symptoms; DAN-PSS = Danish Prostatic Symptom Score; ICS-BPH = International Continence Society-Benign Prostatic Hyperplasia; IPSS = International Prostate Symptom Score; LUTS = lower urinary tract symptoms; M = men; NA = not applicable; W = women.

* Includes population-based studies reporting on specific bother of more than five LUTS, identified by PubMed search (up to December 18, 2013) with terms *bother* combined with *lower urinary tract symptoms* or *LUTS* or *urinary symptoms*. Among symptomatic subjects, the proportion of individuals with bother for each LUTS was calculated to determine the individual level of bother of each symptom. To determine the most prevalent bother at the population level, the prevalence of respondents with bother was calculated for each symptom.

a The study defined nocturia as more than two times per night.

b This information was not reported in the paper but was calculated by us.

c Urinary urgency (population level) and urgency urinary incontinence (individual level) were the most bothersome when both genders were combined.

d According to the article, terminal dribble was assessed by using the question: “Would you ever drip a little bit of urine just as you are leaving?” However, we consider this as postmicturition dribble.

### 4.3 Implications of the findings

From the population perspective, this study identifies urgency as the most pressing public health priority across both genders, a situation reflected in the current major interest in so-called overactive bladder syndrome [29]. For women, SUI is and should be a major priority, with a high prevalence of bothersome symptoms (12% of women). Notably, 7.5% of all women reported UUI with moderate or major bother, and 6% reported nocturia with moderate or major bother. In contrast, for men most current interest focuses on storage symptoms, and although both bothersome urgency and nocturia were highly prevalent (6.8% and 6.0% of men, respectively), these data suggest that postmicturition dribble (7.7% of men) may be an overlooked area for investigation and funding.

Among symptomatic individuals in both genders, we found that UUI was the symptom most likely to cause moderate or major bother. This is consistent with previous studies that suggest substantial impacts of UUI on quality of life and strong associations with anxiety and depression [3]. Clinicians should be aware of the differing impacts of each subtype of incontinence for women, and question patients carefully regarding the extent of bother, managing patients according to their responses.

Overall treatment compliance with many medications for benign prostatic hyperplasia is suboptimal and especially poor for medications for overactive bladder syndrome, even in comparison with long-term medications for hypercholesterolemia or osteoporosis [30], [31]. We found that only a minority of men or women who reported any symptom had substantial bother. The high prevalence of these symptoms, therefore, does not imply large unmet demand for therapy. Our results suggest that most LUTS are well tolerated across ages and genders, partly probably due to their fluctuating nature [32], [33], and therefore they highlight the importance of bother assessment in clinical practice, especially before invasive diagnostics or initiation of therapy.

## 5 Conclusions

Urinary urgency is the most prevalent bothersome LUTS at a population level, but of individuals with symptoms, UUI is most likely to be rated as bothersome. We observed substantial differences in the frequency of symptoms in men and women, but for symptomatic subjects, the frequency of at least moderate bother was similar.

  ***Author contributions:*** Kari A.O. Tikkinen had full access to all the data in the study and takes responsibility for the integrity of the data and the accuracy of the data analysis.  

*Study concept and design:* Tammela, Auvinen, Tikkinen.

*Acquisition of data:* Tikkinen.

*Analysis and interpretation of the data:* Agarwal, Eryuzlu, Cartwright, Thorlund, Guyatt, Tikkinen.

*Drafting of the manuscript:* Agarwal, Eryuzlu, Cartwright, Tikkinen.

*Critical revision of the article for important intellectual content:* Cartwright, Guyatt, Auvinen, Tikkinen.

*Statistical analysis:* Agarwal, Eryuzlu, Thorlund, Tikkinen.

*Obtaining of funding:* Tammela, Tikkinen.

*Administrative, technical, or material support:* None.

*Supervision:* Cartwright, Tikkinen.

*Other:* None.  

***Financial disclosures:*** Kari A.O. Tikkinen certifies that all conflicts of interest, including specific financial interests and relationships and affiliations relevant to the subject matter or materials discussed in the manuscript (eg, employment/affiliation, grants or funding, consultancies, honoraria, stock ownership or options, expert testimony, royalties, or patents filed, received, or pending), are the following: Teuvo L.J. Tammela is a consultant for Astellas, GSK, and Pfizer.  

***Funding/Support and role of the sponsor:*** This study was funded by unrestricted grants from the Competitive Research Funding of the Pirkanmaa Hospital District and Pfizer Inc. Rufus Cartwright's work was funded by a Research Training Fellowship from the UK Medical Research Council. Kari A.O. Tikkinen's work was funded by unrestricted grants from the Finnish Cultural Foundation and the Finnish Medical Society. The funding sources had no role in the design and conduct of the study; collection, management, analysis, and interpretation of the data; and preparation, review, or approval of the manuscript. The authors’ work was independent of the funders.
